# Field Manipulations in On-Chip Micro/Nanoscale Lasers Based on Colloid Nanocrystals

**DOI:** 10.3390/nano13233069

**Published:** 2023-12-03

**Authors:** Yazhou Gu, Zhengmei Yang, Zhitong Li

**Affiliations:** State Key Laboratory of Information Photonics and Optical Communications, School of Science, Beijing University of Posts and Telecommunications, Beijing 100876, China; yzgu@bupt.edu.cn

**Keywords:** colloidal quantum dot, on-chip lasers, field manipulation

## Abstract

Owning to merits such as bandgap tunability, solution processability, large absorption coefficients, and high photoluminescence quantum yields, colloidal quantum dots (CQDs) emerged as a promising gain material to make on-chip micro/nanoscale lasers with high silicon compatibility. In this paper, we review the recent progress in CQD on-chip micro/nanoscale lasers, with a special focus on the physical properties achieved through field manipulation schemes in different types of cavities. Key aspects include manipulating and engineering wavelength, polarization, and direction as well as coupling and light extraction. Finally, we give our prospects for future research directions toward the integration of robust CQD nano/microscale lasers with photonic integrated circuits.

## 1. Introduction

Photonic integrated circuits (PICs) [1,2,3] are a type of integrated circuit that utilizes light (photons) for transmitting and processing information, compared to conventional integrated circuits (ICs) using electrons. They allow for the integration of various optical components, including waveguides, modulators, detectors, and filters, onto a single chip. This integration offers numerous advantages such as high bandwidth, low power consumption, and compact size, surpassing traditional ICs that rely solely on electrical signals [4,5]. On-chip photonic light sources (lasers) are a heart component of PICs that can efficiently provide light signals for optical communication and information processing compared with their off-chip counterparts. These lasers are typically directly integrated into the chip [6], eliminating coupling losses and facilitating seamless integration with other optical components.

In recent decades, research on on-chip lasers has focused on exploring field manipulations from different cavity designs. Early explorations of microcavity lasers utilized structures such as Fabry-Pérot (FP) cavities [7,8], distributed feedback (DFB) cavities [9,10,11,12,13], and distributed Bragg reflector (DBR) cavities [14,15,16,17,18]. In the pursuit of miniaturization with a focus on cavity or mode sizes, various solutions such as whispering gallery mode (WGM) lasers [19], photonic crystal (PhC) lasers [20], and plasmonic nanolasers [21] have been developed. FP cavities offer high-Q factors and precise gain material positioning but require precise layer thickness control. DFB cavities achieve high-Q factors and single-mode operation but have limited mode selectivity and require precise grating fabrication. DBR cavities provide high-Q factors and single-mode emission but require more layers, increasing footprint and fabrication complexity. WGM lasers offer high-Q factors, low threshold powers, and narrow linewidths but face challenges in achieving single-mode operation and directional emission. PhC lasers provide high-Q factors, low threshold powers, and strong light confinement but require precise design and fabrication. Plasmonic nanolasers offer an ultra-compact size and high-speed modulation but suffer from high Ohmic losses resulting from metal constituents. Therefore, making a cavity design should not only satisfy the specific requirements but also balance the trade-offs among all the designs.

The choice of gain material is crucial for on-chip lasers as it directly impacts their integration capability, optoelectronic performance, and wavelength adaptability. It significantly influences the laser’s overall performance and suitability for specific applications. Different material platforms, such as III–V semiconductors (e.g., gallium arsenide, indium phosphide) [22,23,24], perovskites [12,25,26,27], and colloid nanocrystals [28,29,30], have been explored as gain materials. Colloidal quantum dots (CQDs) are the assembly of nanoscale crystals, which, as a whole, can behave as bulk semiconducting materials [31,32,33]. By tailoring and controlling the nanocrystal size, shape, and composition, CQDs offer numerous advantages and versatility for fabricating micro/nanoscale optical and optoelectronic devices. Unlike epitaxially grown semiconductors, the solution-processable property of CQDs makes them compatible with almost all types of substrates and cavities [34,35,36,37]. Their size-tunable bandgap enables precise control over the emission wavelength, while their high absorption cross section ensures efficient photon absorption. Benefiting from the solution-processable property, CQDs exhibit excellent adaptations and compatibility with different nanostructures and substrates.

CQD suspensions enable the fabrication of devices through solution-based methods such as spin-coating and inkjet printing, facilitating the development of cost-effective lasers [38,39,40]. The large gap between the atomic-like electronic states of CQDs prevents the thermal depopulation of the light-emitting band-edge levels. This results in a reduction in lasing thresholds when compared to bulk materials [41]. Additionally, the large energy spacing between electron levels in strongly confined quantum dots may result in temperature-insensitive optical gain, a highly desirable characteristic for laser applications [42,43,44]. Colloidal nanocrystals have also emerged as promising gain materials for amplifiers [45], photodetectors [46], LEDs [47], and other optoelectronic devices [48,49]. Among all the CQD compositions, CdSe and PbS manifest tunable bandgaps covering the entire visible and near-infrared frequency. Therefore, these two kinds of CQDs are the most popular ones to make on-chip micro/nanoscale lasers. In this paper, we reviewed the recent progress of CQD-based on-chip micro/nanoscale lasers, with a particular concentration on key physical properties such as wavelength, polarization, direction, and coupling arising from the field manipulations in cavities. Finally, we provide our prospects for future research directions.

## 2. Review

### 2.1. Wavelength Manipulation

Wavelength tunability is an essential characteristic of on-chip lasers. Tunable lasers play a significant role in fields such as spectroscopy [50], optical communication [51], and biomedical imaging [52,53,54] by promoting integration with other optical components and providing dynamic control over laser output [55,56,57]. CQDs offer a tunable bandgap, which can be easily achieved by controlling QD size and density [58,59]. The cavity mode wavelength can also be adjusted to accommodate this by changing the parameters.

**Figure 1:**
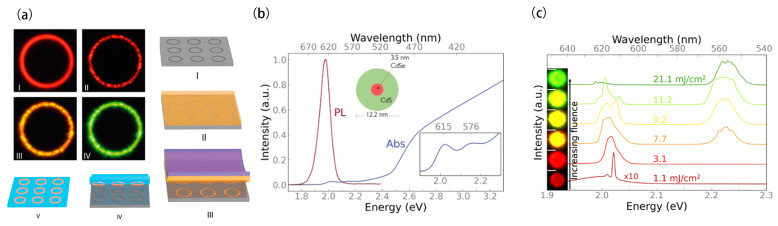
(**a**) Color switching in CQD ring lasers. The absorption and PL spectra, schematic overview of the fabrication process, and Ture-color photographs. (**b**) Tuned emission spectra from different SPs can be repeatedly tuned by cycling the excitation fluence. Reprinted with permission from Ref. [60]. Copyright 2017, ACS; Reprinted with permission from Ref. [61]. Copyright 2023, ACS.

Lasing tunability can be achieved by controlling the optical pumping fluence. In 2018, Feber et al. [60] developed high-quality quantum-dot ring lasers with color-switching properties. These ring resonators consisted of CdSe/CdS/ZnS core/shell/shell CQDs, which were fabricated using a template stripping process (Figure 1a). The CdSe core had a diameter of 3.2 nm and was surrounded by a 12-monolayer-thick CdS shell, further encapsulated by a 2-monolayer-thick ZnS shell. The silicon ring template was fabricated through standard electron-beam lithography and reactive-ion etching, with a diameter of 5 μm and a width of 500 nm. The QDs were then spin-coated to fill the template, followed by a strip-off process to clean the surface. The resulting QD rings had quality factors up to 2500. When the excitation power exceeded the lasing threshold (25 μJ/cm^2^), red lasing with a peak wavelength at 610 nm was observed from the CdSe core. At higher pump powers above 250 μJ/cm^2^, green lasing appeared from the CdS shell while suppressing the red lasing. The phenomenon of color conversion was attributed to the green-like simulated emission rate of the CdS shell exceeding the rate at which excitons localized to the CdSe core, thereby preventing core population inversion. The red–green lasers generated through this design could directly modulate data signals for wavelength division multiplexing in visible light communication systems.

Similar concepts have also been applied in microsphere cavities, enabling tunable continuous-wave lasing. In 2023, Neuhaus et al. [61] proposed WGM lasing from microscale superparticles (SPs) (Figure 1b). The SPs were embedded with thick-shelled CdSe/CdS core/shell CQDs with 3.5 nm diameter cores and 4.4 nm thick shells. Using a high-fluence light soaking protocol reduced the lasing mode blue shift to 1.7 ± 0.5 meV, with champion SPs exhibiting shifts below 0.5 meV. As shown in Figure 1c, real-space images of the lasing QD SPs, displayed in the insets, correspond to the respective excitation fluences. Light-soaking QD SPs under high-fluence provided spectral stability to their laser mode, enhancing the feasibility of the QD SP micro-laser architecture. By increasing the pump flux, optical control of red and green lasers was achieved, demonstrating reversible tuning capabilities. These advantages make the QD SP laser a low-cost, robust, solution-processable, and reusable light source.

**Figure 2:**
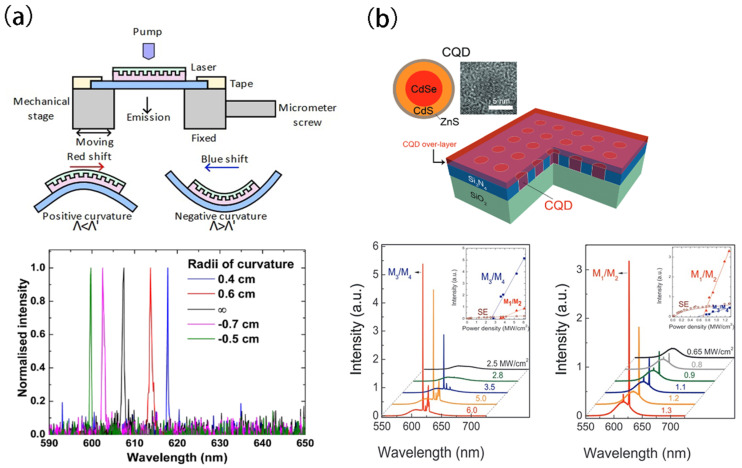
(**a**) QD-stimulated emission schematic and spectra of ASE and laser through grating period tuning. (**b**) Schematic of the CQD-PhC band-edge laser structure and lasing properties of CQD-PhC band-edge lasers. Reprinted with permission from Ref. [64]. Copyright 2014, AIP; Reprinted with permission from Ref. [29]. Copyright 2016, RSC.

Tunable lasing can also be achieved by implementing CQDs into a DFB laser cavity [62,63]. In 2014, Foucher et al. [64] presented a wavelength-tunable CQD laser on an ultra-thin flexible glass substrate (Figure 2a). The device consisted of a DFB cavity, combining a colloidal quantum dot gain film with a grating-patterned polymeric underlayer, all on a 30 μm thick glass sheet. The total thickness of the structure was 75 μm. The hybrid laser showed an average threshold fluence of 450 ± 80 μJ/cm^2^ (for 5 ns excitation pulses) at an emitting wavelength of 607 nm. By mechanically bending the thin glass substrate, the emission wavelength could be tuned continuously over an 18 nm range from 600 nm to 618 nm. Such wavelength shift during bending was attributed to the interaction between the curvature radius of the glass substrate and the overall mechanical performance of the layered laser structure. The confirmed correlation between glass substrate curvature and emission wavelength provided a novel avenue to design tunable lasers. These lasers are highly suitable for applications in bio-sensing, fiber-based, and free-space optical communication as well as in laser radar technology.

PhC lasers, due to their compact size and low operation power, have become an ideal candidate for designing on-chip lasers [65,66,67,68,69]. The tunable bandgap and high quantum yield of CQD material can improve PhC lasing performance in, for example, threshold and output power. In 2016, Chang et al. [29] observed room-temperature lasing from a novel CQD-PhC band-edge laser. The entire PhC cavity consisted of core–shell–shell CdSe/CdS/ZnS core/shell/shell CQDs embedded in a passive 2D square lattice Si_3_N_4_ PhC backbone. The lattice constant and air hole diameter were 255 nm (a) and 140 nm (2r), respectively. The waveguide structure consisted of a Si3N4 slab with a CQD overlay, sandwiched between fused silica and air (Figure 2b). To characterize the lasing phenomenon, micro-photoluminescence (micro-PL) experiments were conducted at room temperature under ambient conditions. When pumped by a 532 nm picosecond pulsed laser, two samples were analyzed—T-80 with an 80 nm CQD overlay and T-130 with a 130 nm overlay. Emission spectra at varying pump powers revealed two sharp lasing modes for both samples, separated by Δλ ≈ 11 nm. These corresponded to high- and low-index contrast band-edge modes. The laser threshold was relatively high at ~1000 μJ/cm^2^ due to limited optical gain from the thin CQD overlay. However, increasing the overlay thickness to 130 nm significantly reduced the threshold to ~300 μJ/cm^2^, around four times lower than that of the T-80 sample. Unlike conventional PhC lasers, this cost-effective fabrication approach enables tailored spatial locations and sizes, making it suitable for high-density photonic integrated circuits. Further optimizations are expected to improve laser performance and mode controllability.

### 2.2. Single-Mode and Multi-Wavelength Manipulation

Single-mode lasing is one of the requirements in PICs for telecommunications. There have been substantial advancements in attaining single-mode lasing in CQD nanolasers, highlighting impressive progress in this domain. In this regard, different approaches and a variety of cavity designs including WGM cavities [70], DFB cavities [71], and vertical cavity surface emitting lasers (VCSELs) [72,73] have been successfully exploited to demonstrate single-mode operation.

**Figure 3:**
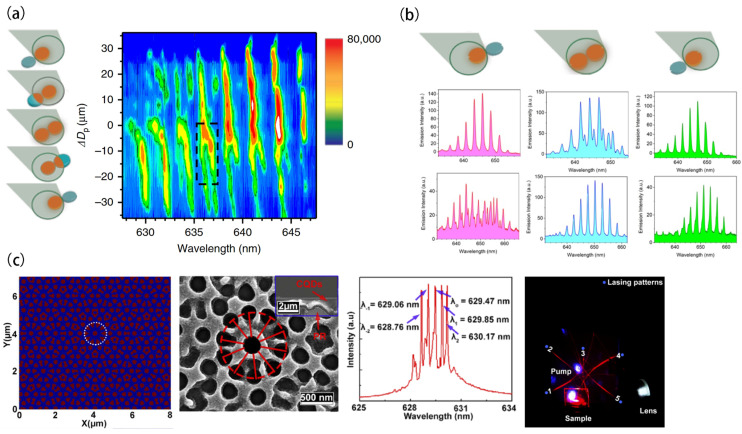
(**a**) Contour maps of emission intensity versus wavelength and relative distance for pumped beam. When microdisk pairs approach the center, mode separation merges. (**b**) Laser emission characteristics of microdisk pairs with and without engineered notches under spatial gain variation. (**c**) The simulation of the interference pattern, a dotted line circle as the base unit. Topography of the 2D photonic quasicrystals. Inset: cross-sectional SEM of the laser device. Multi-wavelength emission spectra, and experimental characterization. The numbers 1, 2, 3, 4, and 5 depict five patterns of DFB lasers with blue circular markings, symmetrically distributed in a two-dimensional space. Reprinted with permission from Ref. [75]. Copyright 2019, Springer Nature; Reprinted with permission from Ref. [76]. Copyright 2019, ACS; Reprinted with permission from Ref. [77]. Copyright 2021, OSA.

Notably, selective PT symmetry breaking can be employed to achieve stable single-mode operation in microring laser resonators. Specifically, PT-symmetric ring pairs enable the systematic enhancement of the maximum attainable gain compared to competing modes. This mode selectivity mechanism remains robust against fabrication inaccuracies and can accommodate active media with wide gain spectra. Furthermore, the occurrence of PT symmetry breaking is solely determined by the relationship between net gain and coupling, making the proposed arrangement inherently self-adapting. PT-symmetric configurations offer superior efficiency performance and are particularly well-suited to controlling longitudinal modes in microring resonators, a previously challenging task. This concept has also been adapted for CQD microlasers. In 2017, Vardeny’s group designed and fabricated a parity-time (PT)-symmetric coupled microdisk laser using CdSe/ZnS core/shell CQDs, with gain in one cavity and loss in the other [74]. In the experiment, mode splitting was observed when gain and loss were balanced in the coupled cavities. Building on this, in 2019, the same group studied coupled microdisk cavities of crosslinked CdSe/Cd_1−x_Zn_x_Se_1−y_S_y_ core/alloy shell CQDs made by scalable photolithography [75]. Active-pump interactions in laser microcavity pairs A and B showed greater resilience to mode splitting. The researchers observed changes in the laser emission modes by gradually varying the position of the pulsed-pump beam in the microdisk pair. Clear mode splitting was observed when only microdisk A was excited, meaning only A behaved as a gain cavity. Subsequently, as the microdisk pair moved toward the center of the pulsed-pump beam and gain was introduced into the other microdisk B, localized cavity modes gradually approached each other in frequency, ultimately merging near the center but not completely overlapping. Figure 3a depicts the spectral results in the PT-symmetric configuration, where only one microdisk was pumped while the other remained unpumped with limited losses. Due to a relatively large gain/loss contrast, this configuration determined the emission modes in the pumped disk, thus recovering the expected free spectral range. The emission spectra of the coupled microdisk laser pair exhibit merged split modes as the pump beam was moved, with several mode pairs at 635–645 nm merging when the disks approached the beam center, with minimized gain/loss contrast. Later in 2019, the same authors explored the same CQD PT-symmetric coupled microdisks but with engineered notches on both of the disks that induced mode interactions that could enable emission spectral control [76]. By incorporating engineered notches into a microdisk laser, the splitting between clockwise (CW) and counterclockwise (CCW) modes could be effectively reduced, resulting in the enhanced directionality of the laser emission. When modulating the contrast between external gain and loss in microdisk pairs with large-sized notches, the inter-disk coupling broke the PT symmetry and led to mode splitting, which was suppressed in this case. On the other hand, microdisk pairs with small-sized notches and strong coupling exhibited anisotropy, where the merging of split modes and the breaking of degenerate modes occurred. Experiments were conducted on cavities with 200 nm and 1000 nm deep notches, respectively. Localized emission occurred when a disk pair was pumped at the edge. Balanced central pumping of both disks enabled mode merging (Figure 3b). Microdisk pairs with 200 nm notches showed residual splitting under balanced pumping. However, disk pairs with deeper notches (1000 nm) could more effectively suppress mode splitting and enhance directionality. The notches mediated backward propagation via coupled mode theory, providing spectral purity. Breaking PT symmetry by differential pumping yielded splitting, while small notches produced merging via anisotropic coupling. Spatial gain/loss modulation eliminated local parasitic modes through a merging of exceptional points in the multimode system, which was attributed to splitting from coupling interactions. Overall, introducing PT-symmetric models in coupled CQD microlasers with tailoring gain/loss raises novel spectral properties in lasing. The QD gain and photolithographic patterning offer an integrated photonics platform to advance laser technologies through emerging non-Hermitian photonics concepts.

Not limited by single-mode lasing, multi-wavelength lasing in CQD nanolasers has also been explored. The use of CQDs in 2D quasicrystal lasers provides enhanced efficiency in coherent lasing through radiation feedback, high-quality optical modes, and long-range rotational symmetry. In 2022, Hayat et al. [77] employed CdSe CQDs in a 2D holographic photonic quasicrystal structure to demonstrate multi-wavelength laser emission (Figure 3c). The 2D quasicrystal structure was designed with 10-fold rotational symmetry to support multiple modes through a coupling mode analytical model. In an experiment, the entire structure was fabricated through a holographic lithography process. It exhibited exceptional energy efficiency with a remarkably low lasing threshold of 26.5 μJ/cm^2^ under optical pumping from a 343 nm wavelength pulsed semiconductor laser. When the pumping power density reached 76.4 μJ/cm^2^, five distinct lasing modes appeared simultaneously, separated by approximately 0.8 nm, with a predominant central wavelength of 629.47 nm. Thanks to the quasicrystal design, lasing modes exhibited a star-shaped emission pattern. The amalgamation of CQDs and holographic lithography represents a potent strategy for achieving efficient and customized lasing within photonic quasicrystals. In summary, this groundbreaking accomplishment of realizing multi-wavelength lasing in a 2D CQD photonic quasicrystal has substantially furthered our comprehension of laser behavior in complex cavity structures.

### 2.3. Polarization Manipulation

The development of on-chip micro/nanoscale lasers heavily relies on the manipulation of the polarization state of light [78]. Polarized beams have applications in multiple areas such as high-resolution imaging [79], nanolithography [80], and high-density optical memories [81]. In this regard, different approaches have been explored to manipulate emission polarization from CQD nanolasers.

**Figure 4:**
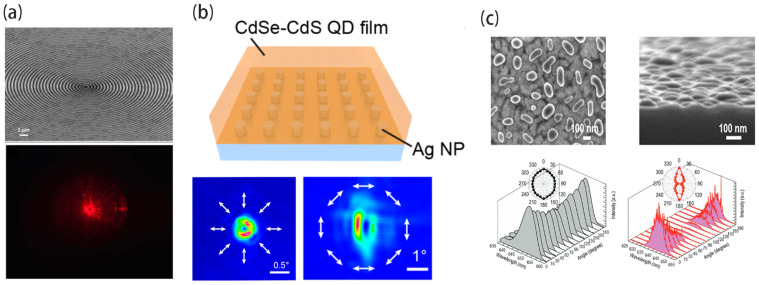
(**a**) High spatial coherent laser and azimuthally polarized far-field radiation image. (**b**) Radial and azimuthal polarized laser emissions are achieved by adjusting the thickness of CQD film for W_TE_-SLR/W_TM_-SLR mode switching. Schematic diagram of CQDs on top of 2D Ag NPs and the far-field pattern of W_TM_-SLR/W_TE_-SLR modes. (**c**) Polarization anisotropic emission tuned by the orientations of Ag NPs: cross-sectional scanning electron microscope (SEM) images, the lasing spectra measured under different polarizer angles, and a schematic diagram of the experiment. Reprinted with permission from Ref. [84]. Copyright 2016, ACS; Reprinted with permission from Ref. [93]. Copyright 2020, ACS; Reprinted with permission from Ref. [94]. Copyright 2016, John Wiley & Sons.

Azimuthally and radially polarized light can generate strong axial optical forces, making them advantageous for optical trapping applications [82,83]. These properties have also been investigated in CQD nanolasers. In 2016, Gao et al. [84] designed a CQD laser by incorporating CdSe/CdZnS/ZnS core/shell/shell CQDs into concentric circular Bragg gratings comprising 250 periodic grooves with 376 nm spacing. This enabled the development of a CQD-based circular grating distributed feedback (CG-DFB) laser (Figure 4a). The group discovered that the lasing wavelength could be tuned by modulating the CQD layer thickness. A breakthrough was the observation of azimuthal polarization, distinct from conventional linear or radial modes, under 183 μJ/cm^2^ pulsed optical pumping. The azimuthal polarization arose from the cylindrical boundary conditions imposed by the circular grating geometry. Analysis of the far-field radiation pattern showed a dim central spot, attributable to the cylindrical boundary conditions and azimuthal polarization. The emitted light’s electric field amplitude was zero at the center and normal at the device plane. Varying the analyzer orientation produced changes in the far-field radiation, confirming the azimuthal nature. Given their surface-normal emission, low laser threshold, single-mode laser operation, circular beam profile, azimuthal polarization, and high spatial coherence, and the unique optical properties of CQDs, CQD-CG-DFB lasers hold immense potential for a wide range of applications in fields such as displays, lighting, photonic circuits, and high-power lasers.

Nanoscale metallic particles arranged in a two-dimensional lattice can support localized surface plasmon resonances (LSPRs) that interact with propagating optical modes, generating high-quality surface lattice resonances (SLRs) [85,86,87,88,89]. SLRs offer strong field confinement near the plasmonic particles and interact with photonic modes. By manipulating the band structure of SLR modes through adjustments in the refractive index [90], light polarization, and lattice geometry [91], nanoscale lasing with switchable, multimodal emission [92] has been achieved. In 2020, Guan et al. [93] explored QD-plasmon nanolasers to achieve a switch between radially and azimuthally polarized light. A periodic array of Ag nanoparticles (NPs) (with a spacing of 400 nm, diameter of 70 nm, and height of 60 nm) was coated with CdSe/CdS core/shell QDs (Figure 4b). Conformal coating of QD films on Ag lattices enabled the formation of hybrid waveguide-surface lattice resonance (W-SLR) modes. QD-plasmon nanolasers were optically pumped at room temperature using a 400 nm femtosecond laser. The far-field emission pattern of the on-chip laser exhibited a characteristic doughnut shape, as observed and measured using a CCD beam profiler. By analyzing the polarization of the doughnut-shaped emission using a rotating linear polarizer, radially polarized laser emission was confirmed. The resulting images showed two lobes that followed the rotation of the polarizer, indicating radially polarized laser emissions from the QDs. By tuning the thickness of the QD film to control the overlap between the W_TE_-SLR/W_TM_-SLR modes and QD emissions, switching between radially and azimuthally polarized directional laser emissions could be achieved. The hybridization of waveguide modes and SLR modes allowed for the generation of laser beams with controlled polarization patterns. The nanoscale energy transfer control between QD and W-SLR modes holds promise for hybrid QD-plasmon systems in quantum communication and information applications.

Except for the observation of radially and azimuthal polarization, polarization anisotropy behavior can be achieved by manipulating the fields in NP. In 2017, Yao et al. [94] applied a technique involving the deposition of a thin film containing CdSe/ZnS core/shell CQDs onto glass substrates covered with elliptical Ag NPs (Figure 4c). The elliptical shape of the silver NPs played a crucial role in lasing with optical anisotropy. Through FDTD simulations, they determined that the orientation of the emitted light’s oscillating electric field relative to the long axis of the Ag NPs strongly influenced the coupling of excitons with localized surface plasmons (LSPs). When the electric field of the emitted light was parallel to the long axis, strong exciton-LSP coupling occurred. The elliptical silver NPs selectively excited LSP resonances by coupling with the oscillating electric field parallel to their major axes, thus playing a critical role in the system. This significant induced effect of NPs on light polarization was observed. Under polarized optical pumping, the intensity of the laser spectrum in the CQD thin film with elliptical Ag NPs was sensitive to the transmission axis of the polarizer. Despite significant variations in the polarizer’s transmission axis, the oscillation modes of the laser spectrum remained in stable positions. This indicated that in systems with elliptical Ag NPs, most of the laser modes have electric fields oscillating perpendicular to the incident plane. The high optical polarization anisotropy results in polar patterns with maximum laser intensity at both ends (*θ* = 0° and 180°). The relative orientation between the emitted light and Ag NPs, along with multiple scattering events, collectively led to the strong optical anisotropy observed in the system. By measuring a series of laser spectra at different polarizer angles, they comprehensively analyzed the polarization anisotropic characteristics associated with the emission. The system demonstrated CQD-based random lasers and implemented coherent and polarized laser emissions. Further exploration is warranted to explore the applications of this system in areas such as light modulation, display technology, and medical diagnostics.

**Figure 5:**
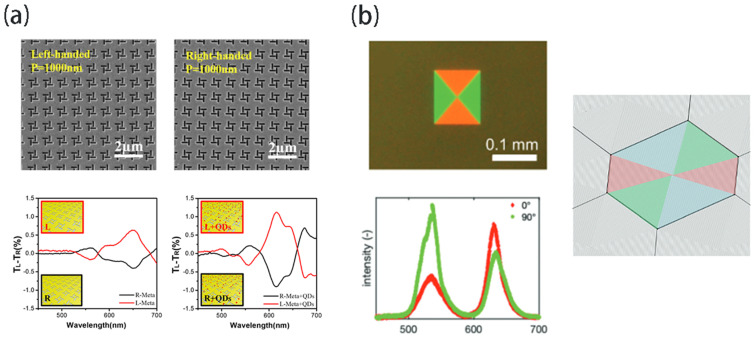
(**a**) Circularly polarized light is generated through a plasma chiral metasurface. The SEM images of left-handed and right-handed chiral metasurfaces and CD spectra and the setup adopted for measuring the luminescent circular polarizations. (**b**) The linear polarization emission is adjusted by the conditional linear Bragg grating Angle. Fluorescence microscopy image of a concentric rectangular/hexagonal grating and fluorescence spectra taken at the center of the structure in rectangular grating. Reprinted with permission from Ref. [95]. Copyright 2019, John Wiley & Sons; Reprinted with permission from Ref. [96]. Copyright 2022, RSC.

Plasmonic chiral metasurfaces possessing broken inversion symmetry can generate circularly polarized light and exhibit circular dichroism (CD). In 2019, Wang et al. [95] conducted a study where they induced chirality in otherwise achiral CdSe/ZnS core/shell CQDs by integrating them with a plasmonic chiral metasurface. The metasurface comprised a square lattice array (periodicity 1000 nm) of four identical nanoslits (length 400 nm, width 100 nm) milled in a 100 nm thick gold film using focused ion beam (FIB) fabrication (Figure 5a). This coupling between the engineered chiral metasurface and the CQDs yielded significant CD and highly circularly polarized emission of the transmitted light. The observed chiroptical response was attributed to correlations between the CQD absorption band and the metasurface’s chiral resonance. Continuous-wave optical pumping from a 442 nm He-Cd laser was conducted on the sample surface with an incident angle of 45°. Emitted PL was collected in the forward direction, transmitted through a zero-order quarter-wave plate and linear polarizer, spectrally dispersed in a monochromator, and detected on a silicon CCD camera. This study demonstrated the efficacy of plasmonic chiral metasurfaces in manipulating CQD emission chirality, realizing pronounced circular polarization with a degree of 17%. Preferential absorption and emission of circularly polarized light confirmed the coupling mechanism between the metasurface and CQDs. The observed selective absorption and emission of circularly polarized light (CPL) provided evidence that the coupling mechanism between the metasurface and CQDs can serve as an effective platform for generating and absorbing CPL. This has implications for miniaturizing optical systems in areas such as biosensing, bioimaging, and quantum communication.

Linear Bragg gratings can resonantly outcouple and generate linear polarized fluorescent emissions from lasers. De Leo et al. [96] recently demonstrated linear polarized lasing from direct patterning linear Bragg gratings on CQD thin films (Figure 5b). The fabricated gratings enhanced optical outcoupling efficiency through Bragg scattering, yielding bright emissions with linear polarization along the grating orientation. This study utilized rectangular grating structures on films containing red (635 nm)- and green (540 nm)-emitting CdSe/CdZnS core/shell CQDs to achieve polarization-controlled multicolor emission. The grating comprised two orthogonal periodicities (Λ1 = 340 nm, Λ2 = 400 nm) for selective outcoupling enhancement that was normal at the surface. Significant enhancements of 86% for red and 71% for green emissions occurred relative to the non-resonant background. The orthogonal grating orientation associated each color with a distinct linear polarization. Polarization-resolved spectroscopy showed 25% and 50% modulation of the red and green peaks, respectively. Moreover, tunable white light generation was demonstrated using concentric hexagonal gratings aligned to blue (460 nm), green (540 nm), and red (620 nm) CQD emission peaks, where the blue peak used CdSe/CdS core/shell CQDs. The hexagonal arrangement facilitated simultaneous resonant outcoupling enhancement along the surface that was normal for all three colors. Exploiting the polarization dependence on grating axis orientation enabled color ratio manipulation with a linear polarizer. Varying the polarizer angle demonstrated tunable white light emissions with adjustable color temperatures. Customizable trajectories within the CIE color space were achieved by polarizer rotation. Further tuning of the color–space trajectory was possible by modifying the CQD composition or grating periods. Grating nanostructures on multicolor CQD films provides an integrated approach to realize polarization-tailored emission for applications in quantum optics, displays, lighting, and nanophotonics. The CQD emission wavelengths and tunable grating parameters enable customizable spectral shaping and polarization control for integrated photonic devices.

### 2.4. Direction Manipulation

One of the key characteristics of lasers is their high directionality [97]. Highly directional beams have important applications in quantum communication and information processing.

**Figure 6:**
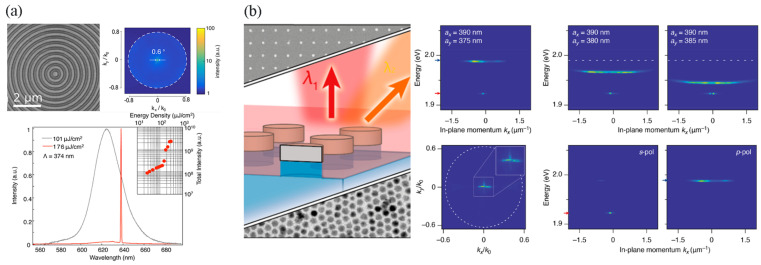
(**a**) The normalized emission spectra are shown below the stimulated emission threshold (gray line) and above it (red line). Inset: The relationship between the total emission intensity and the pump excitation energy density, demonstrating a threshold at 120 μJ/cm^2^. Scanning electron micrographs and a logarithmic intensity scale k-space color map. (**b**) Directional lasing achieved by plasmonic Ag NP arrays. Schematic diagram of dual-wavelength and dual-direction mode excitation. The above-threshold momentum-resolved emission spectra were obtained for rectangular arrays with two different dimensions and polarization-resolved measurements were performed on the lasing emission. Reprinted with permission from Ref. [98]. Copyright 2017, ACS; Reprinted with permission from Ref. [97]. Copyright 2020, ACS.

In on-chip micro/nanoscale lasers, high directionality can be achieved by manipulating fields in various cavities. In 2016, Prins et al. [98] demonstrated highly directional, single-mode lasing with narrow beam widths using high-quality factor concentric grating structures, termed bullseye gratings (Figure 6a). The out-coupling angle was governed by the Bragg condition. When all the radial momentum was matched, the light at a specific wavelength could be vertically emitted from the center of the structure. The condition was $\lambda_{em}=\Lambda n_{eff}$, where $\lambda_{em}$ is the emission wavelength, Λ is the grating period, and $n_{eff}$ is the effective refractive index of the waveguide mode. In an experiment, CdSe/CdZnS core/shell CQD materials were deposited on red-emitting bullseye gratings with a period (Λ) of 374 nm, which was fabricated using a template stripping method. Under optical excitation from a 450 nm wavelength femtosecond laser, single-mode lasing at 638 nm ($n_{eff}$ = 1.7) occurred, with a narrow linewidth of 0.7 nm (2 meV). The directionality was indicated by the beam divergence measurement in the momentum space (k-space). The transition of the spontaneous emission to lasing was accompanied by a significant reduction in beam divergence from approximately 10° (174 mrad) for the spontaneous emission to 0.6° (10 mrad) for the lasing duration. Notably, the lasing thresholds for these structures were consistently below 150 μJ/cm^2^, highlighting their efficient operation. The proposed high-resolution direct patterning approach offers a direct and cost-effective pathway to achieve flexible devices with enhanced directional emission. The stimulated emission from these patterned films, combined with CQD components, has direct technological relevance to optical down-conversion in backlight displays, LEDs, and single-mode surface-emitting lasers.

Plasmonic NP arrays can also be implemented in CQD nanolasers to realize directional emission. In 2020, Winkler et al. [97] integrated CdSe/CdS/ZnS core/shell/shell CQDs with periodic arrays of plasmonic Ag nanodisks to achieve single- and dual-wavelength lasing with high directionality in a visible frequency (Figure 6b). Optical pumping using a 405 nm pulsed laser on the Ag-particle square lattice (a_x,y_ = 390 nm, d = 70 nm) yielded two lasing peaks, separated by ~2 meV, of around 1.93 eV, with divergence angles of 71 mrad along k_y_ and 129 mrad along k_x_ and attributes to second-order Bragg diffraction along the x- and y-lattice directions. Using rectangular lattices, independent tuning of the lasing energies was studied by varying the y-pitch from 375 nm to 385 nm in 5 nm steps while maintaining the x-pitch at 390 nm. As the y-pitch (ay) increased, the high-energy lasing peak redshifted from 1.99 eV to 1.95 eV, while the low-energy peak remained constant at 1.93 eV. The divergence angles also gradually increased to 98 mrad and 205 mrad along k_y_ and k_x_. The lasing emission originated from the transverse electric (TE0) stop gap at k_x_ = 0. At the transverse electric (TE0) and transverse electric (TE1) band intersection points of k_x_, coupling between the TE0 mode with k-vector ${\vec{k}}_{TE0}$ and counterpropagating of the TE1 mode with ${\vec{k}}_{TE1}$ via the second-order grating vector 2$\vec{G}$ led to band opening at k_x_ ≠ 0, yielding off-normal laser emissions. Using a linear polarizer, the s-polarized low-energy peak (x-feedback) and p-polarized high-energy peak (y-feedback) could be switched, confirming dual-wavelength lasing with controllable polarization and directionality.

**Figure 7:**
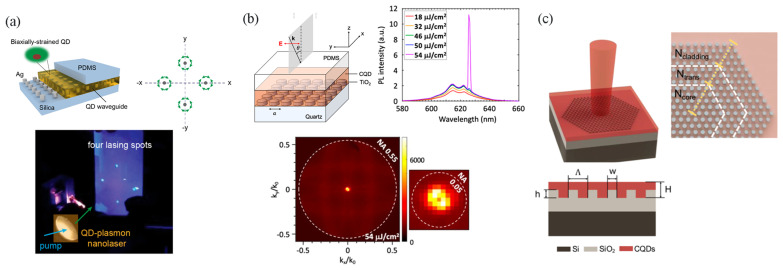
(**a**) Excitation of four-directional laser in a system integrated with plasmonic lattice and CQD waveguide. Charts and photographs of the four laser spots (green circles represent laser beams, arrows indicate polarization direction). (**b**) Room-temperature PhC BIC CQD laser with a low threshold. (**c**) Directional lasing is efficiently excited at the center of the designed quasi-BIC structure. Schematic and longitudinal section diagrams of the heterostructure PhC laser. Reprinted with permission from Ref. [99]. Copyright 2020, ACS; Reprinted with permission from Ref. [104]. Copyright 2021, ACS; Reprinted with permission from Ref. [105]. Copyright 2023, De Gruyter.

In the same year, Jun Guan et al. [99] demonstrated engineered directional lasing in CQD nanolasers by utilizing NP lattices aligned with high-symmetry points in the Brillouin zone. The nanolaser comprised a CdSe/CdS core/shell CQD waveguide layer on a 2D square array of Ag NPs (Figure 7a). The architecture consisted of CdSe/CdS cores (diameter 3.2 nm) and shells (thickness 15.7 nm) conformally coated on square lattices (periodicity a_0_ = 400 nm) of Ag NPs (diameter d = 70 nm, height h = 60 nm) fabricated on a fused silica substrate using scatterless nanolithography techniques. The resulting dense CQD film (thickness 90 nm) functioned as an optical waveguide. To protect the CQD film surface and ensure mechanical robustness, a layer of poly(dimethylsiloxane) (PDMS) was placed on top of the CQD film. Coupling between waveguide modes in the CQD layer and SLRs in the Ag NP lattice yielded W-SLR modes. Leveraging W-SLR feedback near the Δ point enabled lasing at off-normal angles from the CdS shell gain. Four distinct lasing spots were observed corresponding to four Δ points along the x and y lattice axes (Figure 3c). Adjusting the plasmonic lattice periodicity overlapped other high-symmetry points (Γ or M), providing control over different lasing angles by aligning with the CQD emission. Furthermore, increasing the CQD film thickness accessed higher-order W-SLR modes and avoided crossings, expanding the cavity modes available and enabling any desired lasing angle. This research offers valuable insights into the manipulation of interactions between CQDs and optical nanocavities, which holds significant potential for applications in quantum communication and information processing.

Bound states in the continuum (BIC) [100,101,102] are non-decaying resonance modes that were originally conceived in quantum systems but later extended to classical wave frameworks such as photonics, where careful structural designs can create optical BIC modes decoupled from radiation continua through symmetries and index contrast modulation. The ultra-high Q-factors and strong modal confinement of BIC resonances make them ideal for high directivity nanolasers. Recent works have applied BIC concepts to design CQD lasers, leading to demonstrations of room-temperature, low-threshold lasing. QD BIC lasers provide advantages like temperature stability, tunability, and the potential for on-chip integration while facing ongoing challenges to further improve Q-factors and directionality control. PhC lasers support BIC modes and offer several advantages including low thresholds, high efficiency, narrow linewidths, and high Q-factors [103]. The high Q-factors enable enhanced spectral purity and stability. The skin-depth effect facilitates strong interactions with the environment, making BIC lasers suitable for sensing. Additionally, their spatial control allows for the precise manipulation of laser output, demonstrating potential for photonics integration and device applications. In 2021, Wu et al. presented a study on a room-temperature low-threshold BIC laser [104]. The laser comprised a ∼300 nm thick film of CdSe/CdS core/shell CQDs covering a square array of 120 nm tall TiO_2_ cylindrical nanoantennas with a 40 nm gap between cylinders (Figure 7b). The sample was excited using a 355 nm pulsed laser, which exhibited a low threshold of approximately 54 μJ/cm^2^. As a result, a highly directed λ = 626 nm donut laser was emitted in the normal direction. Lasing occurred through the excitation of high Q modes known as BICs originating from TE-polarized first- and second-order slab waveguide modes coupled to the nanoantenna periodicity. Furthermore, the authors distinguished between a BIC mode and a band-edge mode coupled to diffraction, specifically in the context of a second-order DFB mode. They showed that BIC modes were responsible for near-normal lasing directionality in their system. Microscale vortex lasers operating at optical frequencies offer potential advantages in optical communication and quantum information processing.

Directionality can also be achieved by field manipulation in quasi-BIC mode. In 2023, Tang et al. [105] designed hexagonal PhC cavities with 60° rotational symmetry around the z-axis, which supported quasi-BIC modes (Figure 7c). The entire PhC cavity contained three regions: the core, cladding, and transition (heterojunction). Each region contained a hexagonal array of SiO_2_ pillars with a height of 80 nm etched onto a 3 μm thick SiO_2_ layer on a Si substrate. The duty cycle (w/Λ) defined by the ratio of periodicity (Λ) over the diameter (w) of the pillars was the only parameter to adjust the bandgap, which was 0.56 for the core, 0.54 for the transition region, and 0.5 for the cladding. A 130 nm thick film of CdSe/ZnS core/shell CQDs was spin-coated over the pillars, fully covering the SiO_2_ columns and serving as both the optical gain medium and the PhC backbone. This created a mode gap localizing the laser modes near the Γ-point as quasi-bound states in the continuum. The heterogeneous PhC design enabled strong in-plane confinement of the lasing mode while retaining a high-quality factor of up to 2.08 × 10^4^, associated with symmetry-protected BIC states. Tuning just the duty cycle engineered the PhC bandgap across the three regions. Owing to the engineered quasi-BIC nanocavity, the group realized a VCSEL with highly directional emissions within a narrow cone angle of ±1.85° and with a low threshold of 216.75 μJ/cm^2^. While the PhC lacked a full in-plane bandgap, index and mode profile mismatch between the core and higher-order cladding modes suppressed coupling, minimizing out-of-plane radiation losses. This nanophotonic approach will enable scalable, high-efficiency VCSEL arrays for integrated photonic circuits targeting optical communications, sensing, and quantum information applications. The transitional region mitigated abrupt index changes, reducing scattering losses. Overall, the engineered quasi-BIC PhC nanocavity optimized the nanolaser performance with low thresholds and directional emissions.

### 2.5. Coupling Manipulation

Through the careful design of nanostructures, efficient coupling between nanolasers and other nanophotonic components can be achieved. This can minimize energy loss and enhance device performance [106], meeting the integration requirements [107] of on-chip photonic devices.

**Figure 8:**
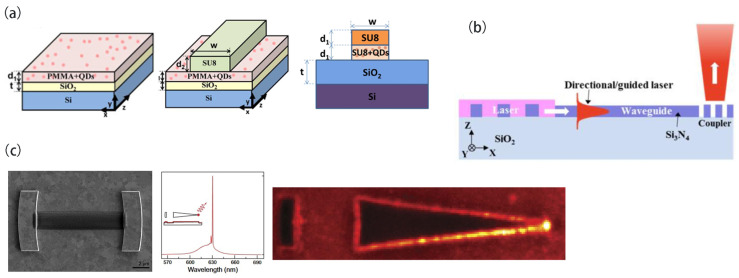
(**a**) The integrated design of planar waveguide with CQD enables directional coupling. Bi-layer waveguiding structures: QD-PMMA/PMMA and QD-PMMA/SU-8. (**b**) A schematic diagram of the on-chip integration of PhC band-edge lasers, waveguides, and output couplers. (**c**) Two Ag block reflectors with extended tapering waveguides. The mode is outcoupled in the conical waveguide. The field energy is concentrated at the nanotip of the spaser. Reprinted with permission from Ref. [109]. Copyright 2013, IEEE; Reprinted with permission from Ref. [110]. Copyright 2016, IEEE; Reprinted with permission from Ref. [111]. Copyright 2017, OSA; Reprinted with permission from Ref. [112]. Copyright 2017, AAAS.

In 2011, the research group headed by J. Martínez-Pastor creatively utilized a planar waveguide composed of CdSe and CdTe (QDs uniformly dispersed in a PMMA layer to achieve directional coupling) [108]. This structure consisted of two PMMA/CdSe (CdTe) and SiO_2_ sol–gel layers on a silicon wafer (Figure 8a). The nanocomposite film could guide the emission of green light (550 nm, CdTe) and orange light (600 nm, CdSe). The optical pumping laser could be coupled to the film through a standard end-face coupling system and could also be directly illuminated on the sample surface, indicating excellent absorption cross-section and waveguiding properties. Building upon this foundation, in 2013, they further expanded the design of CdSe QD waveguides [109]. A planar PMMA layer was placed atop the nanocomposite in their first bilayer structure. This PMMA layer amplified the signal beam’s intensity by capturing the emission radiated from the QDs. Moreover, the pump beam could traverse the cladding without being hindered by QD absorption. Their innovative findings were advanced with the second bilayer structure, where ridge patterns made from a commercially available resist SU-8 were deposited on the nanocomposite active layer. These SU-8 patterns functioned as waveguides for both the pump and signal beams, with minimal absorption losses. In 2016, the research group continued to improve the design of the double-layer waveguide, proposing a bilayer structure composed of an SU8 layer and a PbS QDs layer as well as a two-dimensional ridge waveguide composed of an SU8 cladding layer [110]. This reduced the propagation loss to a total optical loss of 22 dB. By adjusting the power of the pump signal, more than 3 ps of real-time MWP TTD functionality was performed on the 1550 nm optical communication signal over a 25 GHz broadband RF range. In addition, the refractive index of SiO_2_ was close to that of most polymers, enabling the translation of this design to flexible substrates for various applications, such as wavelength tuning through mechanical stretching and bio-recognition for information sensing.

Jung H et al. [111] designed and fabricated a hybrid PhC edge-emitting laser device by exploiting the coupling between waveguides and gratings. This device demonstrated the integration of an active laser component with passive slab waveguides and output couplers on a single Si_3_N_4_ thin-film platform within a simple conceptual PIC (Figure 8b). Si_3_N_4_ and fused silica were chosen as the core waveguide layer and substrate, respectively, due to their high refractive index contrast and extremely low absorption in the visible-to-near-infrared wavelength range. The PhC edge-emitting laser consisted of a 2D array of PhC air holes, while a grating box was used for the output coupler. These structures were formed on a 140 nm thick Si_3_N_4_ layer using successive electron beam lithography (EBL) and reactive ion etching (RIE). The unetched Si_3_N_4_ layer surrounding the structures, in conjunction with the air on top and the silicon dioxide substrate below, formed an asymmetric slab waveguide. The thickness of the Si_3_N_4_ slab waveguide was designed to support only the fundamental guided mode in the vertical direction. Densely packed CdSe/CdS/ZnS core/shell/shell CQDs were then selectively deposited directly on top of the PhC patterns. In these PhCs, the X or M band-edge modes were designed to overlap with the emission wavelength of the CQDs (615 nm), thereby achieving strong interactions between the zero-group-velocity resonant modes and the gain material for lasing. Furthermore, these resonant modes existed below the light line, resulting in in-plane laser emissions with a clear direction (X or M direction) under excitation by femtosecond pulses. Due to the limited number of band-edge modes within the gain range of CQDs, achieving single-mode laser operation on a single chip was easily feasible. The Si_3_N_4_ platform employed in this study is compatible with mature CMOS technology, which holds importance for future developments. The PhC and planar waveguides shared the same coplanar layer, leading to an anticipated high coupling efficiency between them. However, the lack of lateral confinement in these planar waveguides considerably limits the compactness of the PIC and may introduce crosstalk issues in practical information transmission.

In 2017, Stephan et al. [112] experimentally fabricated a metallic cavity with two Ag block reflectors to generate a high Q-factor. They focused on an R = 2L cavity with 10 mm mirror separation and 20 mm apex curvature radius. The gain medium was CdSe/CdS/ZnS core/shell/shell CQDs, which were placed between the two reflectors using electrohydrodynamic NanoDrip printing. Contrary to traditional plasmonic lasers combining gain and cavity by coating the CQDs onto the surface of metallic particles, this design separated the metallic cavity and gain medium, which maximally diminished the metal intrinsic loss. Under optical pulsed excitation, single-mode lasing at a 633 nm wavelength could be observed. When the pump power density was 130 μJ/cm^2^, a minor feature as an early indication of lasing mode appeared in the spectrum. At 250 μJ/cm^2^, the linewidth of this mode sharpened and finally evolved into amplified spontaneous emissions and lasing. They fabricated multiple devices with CQDs at 602 nm, 625 nm, and 633 nm emission wavelengths. For the sake of mode competition, the cavities with 633 nm emissions exhibited a single-mode lasing threshold of 180 μJ/cm^2^, while those with 602 nm and 625 nm emission wavelengths showed multi-mode lasing. To extract the lasing signals, they fabricated an extended tapering waveguide narrowed to a tip of 35 µm in length integrated with one Ag reflector. Under optical excitation, the spaser generated monochromatic plasmonic signals, which were extracted using integrated amplifiers and focused onto a nanotip, producing a strong electromagnetic field (Figure 8c) that could enhance the lasing signals by 10^4^ times. The signal could be further enhanced when the solution-processable CQD material was applied to the extended waveguide. In this scenario, the output of the laser could be amplified after it propagated to the tip, resulting in high field localization of the light. Below the threshold, broadband emissions were observed. Above the threshold, the spatial intensity distribution changed completely as the amplified signal was focused onto the tip, increasing intensity by >1000 times compared to the unamplified signal. The flexibility and broad applicability of this framework may enable spasers to find practical uses in future integrated plasmonic applications.

**Figure 9:**
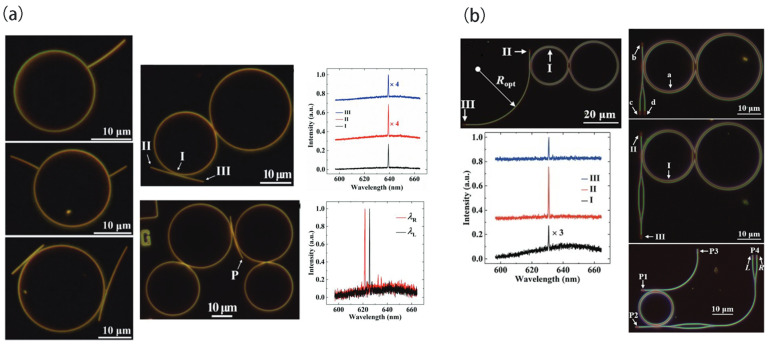
(**a**) Radial and tangential coupling schematic with one-color and two-color single-mode laser emission and coupling in a hybrid structure. (**b**) The laser, waveguide, and functional photonic devices based on CQDs are integrated into a single chip by template-assisted stacking method, which realizes the flexible coupling and routing of optical signals on the chip. Reprinted with permission from Ref. [113]. Copyright 2018, John Wiley & Sons; Reprinted with permission from Ref. [114]. Copyright 2019, RSC.

Inspired by whispering gallery modes in ring resonators, Chen’s group realized the on-chip coupling and routing of laser emissions in waveguide-ring resonators (WRRs) with Ag nanowire waveguides (Figure 9a) [113]. They first deposited a silver nanowire solution on a glass substrate, then spin-coated a PMMA film and accurately located the nanowires using dark-field imaging. Microcavities were patterned at desired positions via electron beam lithography before uniformly depositing CdSe/ZnS core/shell QDs. This deterministic on-chip integration ensured precise alignment between CQD microcavity lasers and Ag nanowires and enabled flexible coupling schemes like tangential, radial, and complex coupling. In tangential coupling, the CQD microcavity center was spaced by ~10.4 μm from the Ag nanowire center, with a gap of ~340 nm, allowing for accurate control of the coupling point, spacing, and gap. Strong laser emissions from the CQD microcavity were observed at a pump fluence of 750 μJ/cm^2^, with output at both nanowire ends without scattering at the coupling point. This yielded a sub-diffraction coherent source (lasing) with a plasmonic mode area of 0.008λ^2^. The lasing remained stable for over 8 months due to the PMMA coating on the nanowires. Besides tangential coupling, radial coupling between a CQD microcavity (D ≈ 20 μm) and Ag nanowire (length ≈ 12 μm) was also demonstrated, with identical laser output wavelengths from the nanowire end. More complex coupling of multiple microcavities and nanowires was realized based on deterministic integration. For instance, tangential coupling of two CQD microcavities (D_1_ ≈ 26 μm, D_2_ ≈ 20 μm) and an Ag nanowire (length ≈ 18 μm) yielded single-mode laser output (λ = 639.2 nm, ∆λ ≈ 0.3 nm) at a pump fluence of 500 μJ/cm^2^, which was coupled to the nanowire with identical emission wavelengths at both ends. This enabled integrated single-mode laser and subwavelength plasmonic waveguides with single-mode sub-diffraction coherent outputs. The flexible configurable integration was extended to demonstrate a dual-color single-mode laser by coupling two single-mode lasers (λ_L_ = 625.4 nm, λ_R_ = 621.6 nm) to a single nanowire. In summary, the stable and deterministic on-chip integration of CQD microcavity lasers and subwavelength plasmonic waveguides was experimentally realized. The implemented strategy can produce integrated lasers with low thresholds, narrow emission linewidths, and sub-diffraction output beams. Such sub-diffraction coherent output could flexibly guide on-chip technology to target plasmonic components like nanoscale filters, splitters, and (de)multiplexers. 

On the other hand, CQD waveguides and ring resonators were fabricated using a template-assisted stacking technique (Figure 9b) [114]. Firstly, a PMMA thin film was spin-coated on a MgF_2_ substrate, followed by high-precision electron beam lithography to create trench patterns in the PMMA film. Then, a CdSe/ZnS core/shell QD solution was drop-cast on the patterned PMMA film, allowing for the uniform distribution of CQDs across the film. Here, CQDs could provide both optical gain and act as a high-index medium, enabling the design of on-chip lasers and other photonic devices. The proposed pattern-assisted stacking facilitated the fabrication of more complex structures owing to the utilization of the same CQD material and EBL systems’ built-in pattern generation capability. The on-chip integration of single-mode CQD lasers with other functional CQD photonic devices was experimentally demonstrated, including bent waveguides, Y-splitters, MZ interferometers, gratings, and optical amplifiers. First, a coupling structure of two WRRs (D1 ≈ 20 μm, D2 ≈ 26 μm) and a bent waveguide was introduced. Single-mode lasing could be achieved in the two coupled WRRs based on the Vernier effect. Under a 116 μJ/cm^2^ pump, only one resonant peak (λ ≈ 630.8 nm) was observed from the emission spectrum of the coupled WRRs. Next, the on-chip assembly of single-mode CQD lasers and functional CQD photonic devices were further demonstrated based on pattern-assisted stacking. A dark-field optical image of another coupling structure composed of two coupled WRRs and a Y-splitter was displayed, which are widely used in PICs for power distribution with minimal insertion loss. The right part showed the coupled WRRs acting as on-chip single-mode laser sources. Under a 165 μJ/cm^2^ pump, lasing occurred in the coupled WRRs, with evident scattered light observed from the gratings at the three ports (positions b, c, and d) of the Y-splitter. The single-mode laser from the coupled WRRs was successfully routed to the Y-splitter. Moreover, an MZ interferometer consisting of two cascaded Y-splitters was also integrated with the single-mode laser experimentally. Owing to the uniformity of the CQD micro/nanostructures, the two arms of the MZ interferometer had nearly equal optical lengths. Under a 192 μJ/cm^2^ pump, a bright spot was observed at the far end of the MZ interferometer (position III) due to constructive interference between the two arm laser signals. Identical single-mode lasing wavelengths were further confirmed by the coupled WRRs (position I) and the ends of the MZ interferometer (positions II and III). A dark-field optical image of an even more complex coupling structure was displayed, including one WRR laser, one MZ interferometer, one Y-splitter, two straight waveguides, two bent waveguides, and five gratings. Under a 260 μJ/cm^2^ pump, a bright spot was observed at each grating, confirming that the laser signal from the WRR laser was coupled and routed to the integrated photonic devices. Therefore, the on-chip integration of CQD lasers (both multimode and single-mode) with other functional CQD photonic devices was successfully demonstrated experimentally using the proposed pattern-assisted stacking approach. Hence, this work may represent an important step toward the realization of photonic integrated circuits.

## 3. Conclusions

Due to the great merits of CQD materials, such as being solution-processable and bandgap-tunable, tremendous research efforts have been devoted to exploring CQD-based on-chip nano/microscale lasers. In this regard, significant properties of lasers such as single-mode operation, wavelength tunability, polarization control, emission extraction, and coupling have been achieved and manipulated in different laser cavities. Wavelength tuning can be achieved by controlling the size and density of the CQDs or by adjusting the parameters of the WGM, DFB, and photonic crystal cavities. For single-mode lasing, those cavities can also be employed. Notably, PT symmetry breaking in coupled micro-ring/disk cavities has also been explored to realize large-area single-mode lasers. Generating different polarization states has been developed by modulating the direction of the DFB grating for directed polarization, using CG-DFB structures for azimuthal polarization, as well as controlling QD thickness to match plasmonic NP arrays for radial or azimuthal polarization light. It is worth noting that a chiral metasurface covering CQDs has been demonstrated to generate circular polarization. Furthermore, highly directional emissions can be achieved through carefully designed circular DFB structures, photonic crystals, and plasmonic structures. Coupling in waveguides has been achieved by integrating photonic crystals, plasmonic structures, and planar waveguides with CQDs. These manipulation schemes are summarized in Table 1.

Future research directions can concentrate on the following aspects: 1. Electrically driven lasing. The ultimate goal for micro/nano on-chip lasers is electrical pumping that can accommodate the integration requirements. Further investigations can focus on exploring approaches to increasing the Q factor and lowering the lasing threshold toward the realization of electrically driven CQD nanolasers. 2. Achieve topological CQD micro/nanoscale lasers. In recent decades, topological photonics has rapidly emerged as a fascinating platform to manipulate electromagnetic waves in a robust way, which can prevent system disorders and defects. Involving the topological phases of matter in CQD nanolaser cavity design can pave an avenue to investigate robust coherent on-chip light sources. We anticipate that these future research directions can not only enrich the field of CQD micro/nano lasers but also stimulate the integration of on-chip lasers in photonic devices in the industry.

## Figures and Tables

**Table 1:** Manipulations of different physical properties.

Physical Property	Cavity Type	CQD Materials	Wavelength (nm)	Threshold (μJ/cm^2^)	Refs
wavelength	WGM	CdSe/CdS/ZnS	610	25	[60]
	WGM	CdSe/CdS	560/620	1200 ± 400	[61]
	DFB	PbS	1553–1649	770	[64]
	DFB	CdSe/ZnS	600–618	450 ± 80	[62]
	DFB	CdSe/CdS/ZnS	613.4–623.2	404 ± 28	[63]
	PhC	CdSe/CdS/ZnS	620/630	1000/300	[29]
single-mode	DFB	CdSe/CdS	532	270	[71]
	VCSEL	CdSe/ZnCdS	560	135	[72]
	VCSEL	CdSe/CdS/ZnS	623	9000	[73]
	WGM	CdSe/Cd_1−x_Zn_x_Se_1−y_S_y_	630–645	29	[75]
polarization	DFB	CdSe/CdZnS/ZnS	626	183	[84]
	W-SLR	CdSe/CdS	635/644	30	[93]
	LSPR	CdSe/ZnS	643	6110	[84]
direction	DFB	CdSe/CdZnS	638	120	[98]
	SLR	CdSe/CdS/ZnS	635.8–652.5	110	[97]
	W-SLR	CdSe/CdS	515	1000	[99]
	PhC	CdSe/CdS	626	54	[104]
	PhC	CdSe/ZnS	588–612	216.75	[105]
outcoupling	WGM	CdSe/ZnS	630.8	116	[114]
	WGM	CdSe/ZnS	629.1/639.2	175/147	[113]
	PhC	CdSe/CdS/ZnS	624	1000	[111]
	planar waveguide	\	\	\	[108,109,110]
	spaser	CdSe/CdS/ZnS	633	180	[112]

## Data Availability

Not applicable.
